# Antimicrobial Activity of Silver-Containing Surgical Dressings in an In vitro Direct Inoculation Simulated Wound Fluid Model Against a Range of Gram-Positive and Gram-Negative Bacteria

**DOI:** 10.1089/sur.2023.155

**Published:** 2023-09-07

**Authors:** Kate Meredith, Lucy Emma Forbes

**Affiliations:** Convatec Limited, Deeside, Flintshire, United Kingdom.

**Keywords:** bacteria, silver, surgical dressings, surgical site infections, surgical wound dressings

## Abstract

**Background:**

Surgical site infections can lead to serious complications and present a huge economic burden. Established wound infections can be difficult to eradicate so preventative measures, including antimicrobial dressings, are advantageous.

**Materials and Methods:**

The antimicrobial activity of an ionic silver, ethylenediaminetetraacetic acid (EDTA) and benzethonium chloride-containing (ISEB) surgical cover dressing (SCD) was compared with two other silver-containing SCDs (silver sulfate and ionic silver carboxymethylcellulose [CMC]) and a non-silver–containing CMC SCD control using an in vitro model. The dressings were tested against a range of gram-positive and gram-negative bacteria found in wound environments, including antibiotic resistant strains, using a direct inoculation simulated wound fluid (SWF) model. Dressings were fully hydrated with SWF and inoculated with a final concentration of 1 × 10^6^ colony forming units (CFU) per 10 microliter of the challenge organisms. Dressings were incubated at 35°C ± 3°C for up to seven days; total viable counts (TVCs) were performed to determine bacterial bioburden.

**Results:**

All challenge organism levels remained high for the CMC SCD control and silver sulfate SCD throughout the test period. A greater than 95% reduction in TVCs was observed by four hours for all challenge organisms for the ISEB SCD, with non-detectable levels (<70 CFU per dressing) reached within 24 hours and sustained throughout the test period. Antimicrobial activity was less rapid with ionic silver CMC SCD, with 9 of 11 challenge organisms reaching undetectable levels within 6 to 72 hours.

**Conclusions:**

A more rapid antimicrobial activity was observed for the ISEB SCD compared with other dressings tested within this in vitro model.

Surgical site infections (SSIs) are defined by the U.S. Centers for Disease Control and Prevention as infections that occur after surgery in the part of the body where the surgery took place.^1^ Surgical site infections often occur within 30 days of surgery, however, late-onset infections related to implanted material can present years post-surgery.^2^ The incidence of SSIs varies with surgery type, with a recent meta-analysis including approximately 0.5 million patients finding a pooled 30-day incidence of 11% for general surgical patients.^3^ Surgical site infections are associated with prolonged hospital stays, increased mortality, re-operation, re-admission, and a reduced quality of life.^4^ The management of SSIs also presents a large financial burden, with a patient with an SSI costing up to an additional $34,000 more than a patient without an SSI in developed countries.^5^

Many healthcare institutions have adopted preventative measures to reduce the incidence of SSIs because of their aforementioned impact. Possible preventative measures include pre-operative bathing, surgical hand preparation, antibiotic prophylaxis, and the use of silver-containing antimicrobial surgical cover dressings (SCDs).^6^ Silver is a broad-spectrum antimicrobial and clinical evidence has suggested that silver SCDs may reduce the incidence of SSI.^7–9^ However, the National Institutes of Health has estimated that 80% of SSIs^2,10^ involve complexes of micro-organisms embedded in a self-produced matrix of extracellular substance called biofilm.^11^ This biofilm complex provides a defense against both the immune response and antimicrobials,^2^ including silver,^12^ making it difficult to eradicate once established. Preventing SSIs and the formation of biofilm is therefore a logical route to improve patient outcomes.

Several foam- or fiber-based SCDs containing silver compounds are commercially available, with differing structures leading to varying levels of antimicrobial activity.^13–15^ Inclusion of a carboxymethylcellulose (CMC) fiber layer, which forms a cohesive gel in contact with wound exudate to sequester and immobilize bacteria, in a silver-containing SCD may potentially reduce the risk of SSIs and protect against biofilm.^16^ A next-generation, silver-containing carboxymethylcellulose dressing (NGAD), which combines antimicrobial silver ions with antibiofilm agents benzethonium chloride (BEC) and ethylenediaminetetraacetic acid (EDTA), was developed to reduce bioburden further. Ethylenediaminetetraacetic acid is a metal chelating agent that has previously been shown to disrupt biofilms,^17^ and BEC is a synthetic quaternary cationic surfactant intended to reduce the surface tension within biofilm.^18,19^ This NGAD has been adapted into an ionic silver, EDTA, and BEC (ISEB)-containing SCD, bringing the additional antibiofilm advantages into use post-surgery to help protect against potential SSIs. Previous in vitro experiments have demonstrated that NGAD agents act synergistically, rapidly decreasing bacterial populations present in biofilm complexes.^18,20^ However, although there is evidence to support these individual features, the antimicrobial properties of the ISEB SCD against planktonic phenotype of bacteria have not yet been compared with other silver-containing SCDs available.

The purpose of this study was to compare the antimicrobial activity of the ISEB SCD with two other commonly used silver-containing SCDs (ionic silver CMC and silver sulfate) and a non-silver CMC SCD control, using an in vitro simulated wound fluid (SWF) model inoculated with known planktonic phenotype cultures of SSI-causing bacteria.

## Materials and Methods

### Test dressings

The dressings used in this study and their silver concentrations are presented in Table 1: the ISEB SCD (AQUACEL Ag Advantage SCD, Convatec, Deeside, UK); a CMC SCD containing silver ions (AQUACEL Ag SCD, Convatec); a foam SCD containing silver sulphate (Mepilex Border Post-Op Ag SCD, Mölnlycke, Gothenburg, Sweden); and a non-antimicrobial CMC SCD, serving as a negative control (AQUACEL SCD, Convatec).

**Table 1.** Surgical Cover Dressings Included in the Study

Surgical cover dressing	Wound contact area	Silver	
		Form	Concentration^a^
ISEB SCD	CMC (Hydrofiber^®^)	Ionic silver	0.29 mg/cm^2^
Ionic silver CMC SCD	CMC (Hydrofiber^®^)	Ionic silver	0.29 mg/cm^2^
Silver sulphate SCD	Silicone adhesion (Safetac^®^)	Silver sulfate	1.2 mg/cm^2^
CMC SCD	CMC (Hydrofiber^®^)	NA	NA

CMC = carboxymethylcellulose; NA = not applicable; ISEB SCD = ionic silver, ethylenediaminetetraacetic acid and benzethonium chloride-containing SCD; surgical cover dressing; SCD = surgical cover dressing.

^a^ Approximate concentration as stated by the manufacturer.

### Microbiological media

Media used during the protocol included SWF (50:50 v/v Maximum Recovery Diluent [MRD; Neogen Corporation, Lansing, MI]: Fetal Bovine Serum [Biowest SAS, Nuaillé, France]), MRD (Neogen), pre-dried Tryptone Soy Agar (TSA) plates (Neogen), and Dey-Engley Neutralizing Broth (DENB; Neogen).

### Challenge organisms

Challenge organisms implicated in SSIs were selected, including both gram-positive and gram-negative species. Strain reference numbers, species names, their antimicrobial resistance properties and other relevant information are shown in Table 2. Strains were supplied by the American Type Culture Collection (Manassas, VA), National Collection of Industrial Food and Marine Bacteria (Aberdeen, UK), and National Collection of Type Cultures (Salisbury, UK).

**Table 2.** Challenge Organisms Used During Testing

Challenge organisms	Strain number	Antibiotic resistance/additional details
Gram-positive		
*Staphylococcus aureus* (CA-MRSA)	ATCC BAA-1556	Methicillin resistant / community acquired strain
* Enterococcus faecalis*	NCTC 12201	Vancomycin resistant
Coagulase-negative *Staphylococcus epidermidis*	NCTC 11047	NA
* Corynebacterium amycolatum*	NCTC 13725	NA
* Staphylococcus aureus*	NCIMB 9518	Methicillin sensitive
Gram-negative		
* Pseudomonas aeruginosa*	NCTC 8506	Streptomycin and cephalosporin resistant
ESBL-resistant *Klebsiella pneumoniae*	NCTC 13465	ESBL-resistant (blaCTX-M group 25 gene)
Carbapenem-resistant *Klebsiella pneumoniae*	NCTC 13439	Carbapenem resistant
* Acinetobacter baumannii*	NCTC 13421	Sequence type 2
* Escherichia coli*	NCIMB 10544	NA
Carbapenem-resistant *Escherichia coli*	NCTC 13919	Carbapenem resistant

ATCC = American Type Culture Collection; CA-MRSA = community-associated methicillin-resistant *Staphylococcus aureus;* CFU = colony forming units; CMC = carboxymethylcellulose; ESBL = extended spectrum β-lactamase; NA = not applicable; NCIMB = National Collection of Industrial Food and Marine Bacteria; NCTC = National Collection of Type Cultures.

### In vitro direct inoculation simulated wound model

#### Preparation of the dressings and inoculation with challenge organisms

**FIG. 1.**
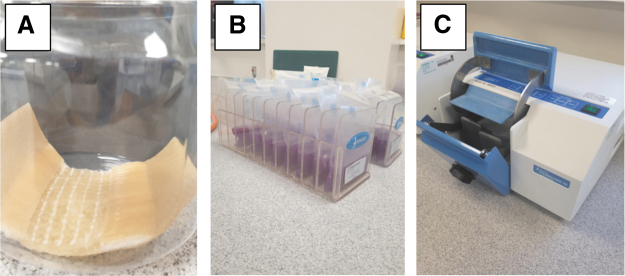
Experimental set-up. Dressings were placed in 300 mL pots, inoculated with bacteria, and fully hydrated with simulated wound fluid; the CMC SCD control is shown (**A**). Dressings were removed and placed in stomacher bags containing Dey-Engley Neutralizing Broth after the incubation period (**B**) before being placed in a laboratory blender (**C**). CMC = carboxymethylcellulose; SCD = surgical cover dressing.

The SCD test and control dressings were aseptically cut into 5 cm lengths, width remaining as stated for the dressing, therefore not altering wound contact area plus adhesive. Dressings were transferred to sterile 300 mL containers. Challenge organisms were prepared by dispersal into MRD to achieve a density of 1 × 10^8^ colony forming units (CFU) per milliliter. A quantitative count was performed to ensure the correct inoculum level. The dressings were then hydrated fully with 5 mL of filtered SWF and inoculated with 10 microliter of challenge organism, making the final bacterial content 1 × 10^6^ CFU per dressing (Fig. 1A). As an additional control, a T_0_ control dressing count was performed on the CMC SCD control dressing, whereby a total viable count (TVC) was performed immediately after inoculation of the dressing, for further confirmation of the initial challenge level.

#### Incubation

Inoculated dressing containers were wrapped in parafilm and incubated at 35°C ± 3°C for 2, 4, 6, 24, 48, 72, 96, or 168 hours (7 days). To ensure that dressing samples incubated for longer time periods remained fully hydrated, 1 mL of simulated wound fluid was added to the dressing containers at 48 hours before being returned to 35°C ± 3°C for the remaining relevant test period. There were three replicates for each dressing incubation period.

#### Total viable count

After the relevant incubation times, the dressings were removed from the incubator and aseptically transferred to a stomacher bag containing 70 mL of Dey-Engley Neutralizing Broth (Fig. 1B) before being homogenized for four minutes on high with a laboratory blender (Fig. 1C) and left to stand at room temperature for two minutes. The resultant suspensions were then serially diluted, inoculated and spread with a sterile L-shaped spreader onto duplicate pre-dried TSA plates. The TSA plates were inverted and incubated at 35°C ± 3°C for at least 48 hours. After the incubation period, the number of CFUs were counted using the most appropriate dilution for each dressing type (i.e., between 25 and 250 CFU per plate) to attain a TVC. The detection limit of the CFU countable in the test was 70 CFU per dressing.

## Results

**FIG. 2.**
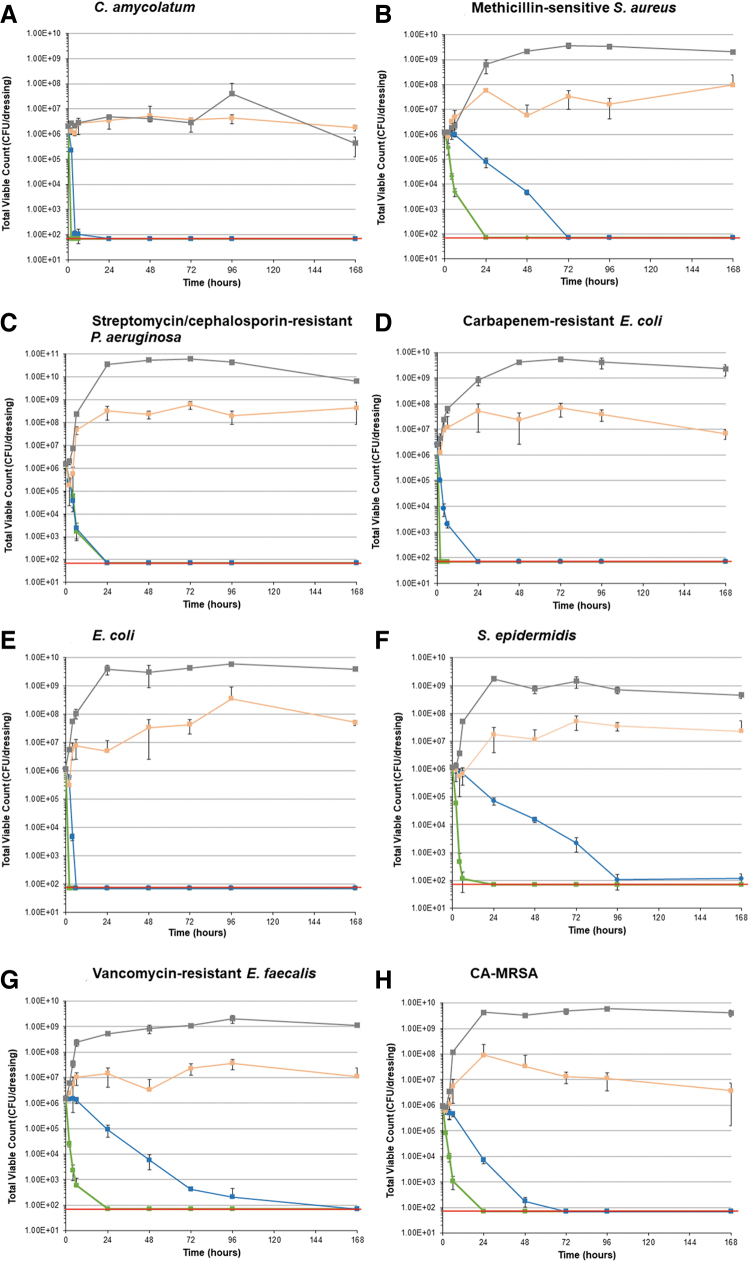

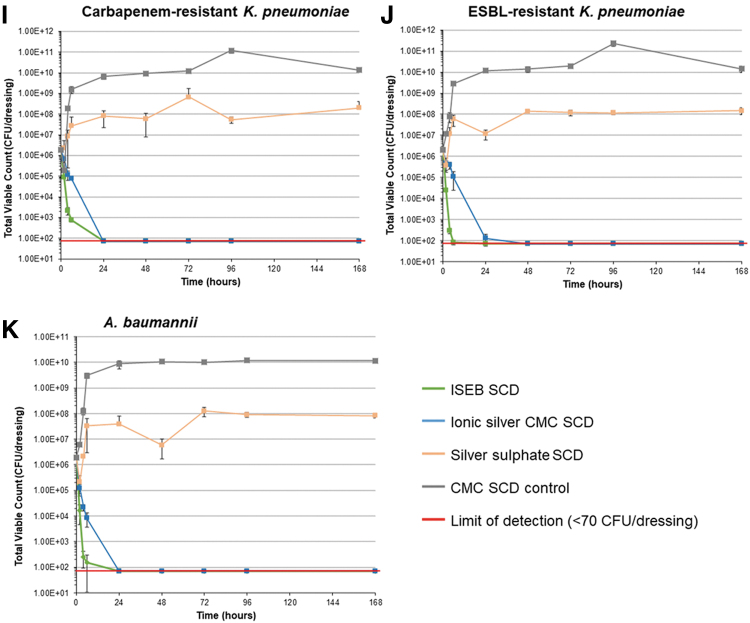
Total viable counts over time for each challenge organism. CA-MRSA = community-associated methicillin-resistant *Staphylococcus aureus*; CFU = colony forming units; CMC = carboxymethylcellulose; ESBL = extended spectrum β-lactamase; ISEB SCD = ionic silver, ethylenediaminetetraacetic acid and benzethonium chloride-containing surgical cover dressing; SCD, surgical cover dressing.

**FIG. 3.**
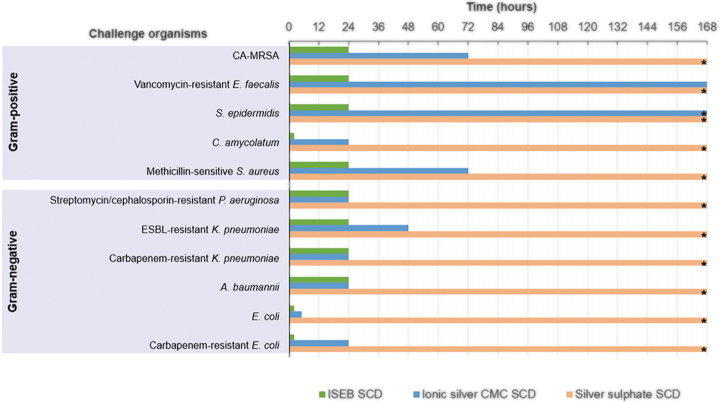
Average incubation time required to reduce total viable bacterial counts of challenge organisms to below detectable limits. The limit of detection was <70 CFU per dressing. *These organisms/dressing combinations did not reach the limit of detection within the seven-day test period. CA-MRSA = community-associated methicillin-resistant *Staphylococcus aureus*; CFU = colony forming units; CMC = carboxymethylcellulose; ESBL = extended spectrum β-lactamase; ISEB SCD = ionic silver, ethylenediaminetetraacetic acid and benzethonium chloride-containing surgical cover dressing; SCD, surgical cover dressing.

The TVC data are presented for all the timepoints tested for each of the challenge organisms in Figures 2 and 3. The CMC SCD control showed a slow increase at the shorter time points (2, 4, and 6 hours) followed by consistently high numbers throughout the remainder of the seven-day test period; average bacterial counts by day 7 remained above 1 × 10^8^ CFU per milliliter for all organisms tested except for *Corynebacterium amycolatum,* which struggled to increase in numbers over the test period but maintained numbers throughout (Fig. 2A). *Corynebacterium amycolatum* is known to grow slowly on routine media so this was an expected finding.^21^

The general antimicrobial efficacy trend for the silver-containing dressings tested showed that the ISEB SCD had the most antimicrobial activity against the inoculated challenged organisms, followed by the ionic silver CMC SCD. A more rapid kill was observed for the ISEB SCD, which reduced TVCs within two hours of dressing inoculation (Fig. 3), with a minimum reduction of more than 95% in all challenge organisms by four hours compared with the initial T_0_ control dressing count. All challenge organisms showed a greater than 99% reduction by four hours, except for methicillin-sensitive *Staphylococcus aureus* and antibiotic-resistant *Pseudomonas aeruginosa,* which had achieved reductions of greater than 98% and 95%, respectively (Figs. 2B and 2C). Bacterial populations were reduced to undetectable levels (<70 CFU per dressing) within two hours for carbapenem resistant *Escherichia coli,* non-resistant *Escherichia coli,* and *Corynebacterium amycolatum* (Figs. 2A, 2D and 2E) and within 24 hours for all other challenge organisms, with sustained antimicrobial activity throughout the remaining test period (7 days).

Bacteria levels for most species incubated with the ionic silver CMC SCD were reduced by 20% to 100% at six hours and more than 93% at 24 hours, reaching undetectable levels (<70 CFU per dressing) within six to 72 hours. Coagulase-negative *Staphylococcus epidermidis* was close to undetectable levels (<1.17 × 10^2^ CFU per dressing) by the end of the test period (day 7; Fig. 2F) whereas vancomycin-resistant *Enterococcus faecalis* reached undetectable levels by day 7 (Fig. 2G).

The silver sulfate SCD was unable to reduce the numbers of any of the challenge organisms inoculated onto the SCD by the end of the seven-day testing period (Fig. 2), although it demonstrated a marginal amount of antimicrobial activity, as shown by a decrease in numbers of some of the challenge organisms at the initial timepoints (e.g. antibiotic resistant *Pseudomonas aeruginosa* at 2 hours; Fig. 2C). This is also suggested by the fact that TVCs did not increase to the levels observed with the CMC SCD control over the seven-day test period.

## Discussion

Overall, the ISEB SCD demonstrated rapid antimicrobial activity, within the dressing, in this in vitro SWF model, reducing a range of bacterial species to undetectable levels within 24 hours, including antibiotic-resistant strains. The rate of kill was more rapid with the ISEB SCD than with ionic silver CMC SCD, despite the only difference being the introduction of the antibiofilm agents (BEC and EDTA) in the ISEB SCD, indicating that the addition of such agents enhances antimicrobial activity. This is consistent with previous studies, although against biofilms, demonstrating that the combination of silver and the additional agents BEC and EDTA produce a synergistic effect on antimicrobial activity.^18,20^

The rapid kill rate of bacteria observed with the ISEB SCD may be particularly beneficial in the prevention of SSIs. Biofilms initially form from planktonic bacteria that colonize on wound surfaces and biofilm forms rapidly.^2^ This biofilm phenotype is difficult to remove once formed because of its recalcitrance to antimicrobial agents and the patient's own immune system.^2^ The ISEB SCD demonstrated antimicrobial activity against the organisms that are known to form biofilm in vivo, consistent with the performance of NGADs in in vitro biofilm models.^18,20,22^ Moreover, the causative organism in 70% to 95% of SSIs are human skin commensals that exist in polymicrobial microbiomes on the skin.^23^ Therefore, the rapid antimicrobial activity and if required, the antibiofilm properties, of the ISEB SCD may be particularly helpful in preventing the development of difficult-to-treat, biofilm-associated SSIs.

A notable finding was the improved antimicrobial activity observed with the ionic silver SCDs compared with the silver sulphate SCD, despite the latter containing a nearly 10-fold higher silver content (Table 1). The differences in dressing efficacy might be attributed to the different wound contact areas: silver sulfate SCD has a silicone wound contact layer (Safetac^®^-Molnlycke, Gothenburg, Sweden) while the ionic silver SCDs use gelling CMC (Hydrofiber^®^-Convatec, Deeside, UK). As previously discussed, CMC is able to immobilize bacteria^16,24^ and may facilitate bacterial contact with the active agents within the dressing. Silicone on the other hand, is impermeable to bacteria^14^ and may prevent such contact. Previous studies have shown that the addition of a silicone layer can hamper antimicrobial activity, preventing the silver content in the dressing to contact and kill bacteria.^14,15^

One study reported that foam dressings with adhesive silicone wound contact layers did not prevent bacterial proliferation, while gelling fiber dressings killed bacteria in a flat bacteria-seeded-agar model.^14^ Another in vitro study evaluating four silver-containing dressings found that bacterial growth with silicone adhesive wound contact layers was considerable compared with dressings with CMC gelling fiber contact layers, which had negligible or absent levels of bacteria despite having five to 11 times lower silver content.^15^ Together with our findings, these studies show the importance of dressing technology and design, and suggest that dressing structure rather than silver content, drives differences in antimicrobial performance. This variation in dressing design could explain the lack of consistent findings in the effectiveness of silver-containing dressings in SSI prevention.^25^

A limitation for this study was that the challenge organisms selected only represent aerobic organisms commonly implicated in SSIs, despite the implication of anaerobic species^27^ and fungi^28^ in SSIs. Additionally, as a general limitation of in vitro work, findings may not be generalizable to the clinical situation; however, the method used is an adapted variation of the American Association of Textile Chemists and Colorists (AATCC) test method 100 (antibacterial finishes on textile materials),^26^ which is often adapted and used to test antimicrobial efficacy of dressings. Although there is evidence to suggest that the NGAD improves healing outcomes in chronic wounds,^29,30^ clinical studies on its use in the prevention of SSI have yet to be conducted. Previously, a randomized controlled trial on the ionic silver CMC SCD found a 10-fold reduction in the incidence of orthopedic SSIs^7^. Therefore, an important future direction is to determine whether the enhanced antimicrobial properties of the ISEB SCD over the ionic silver CMC SCD observed in the current in vitro study, translate into a greater reduction of SSI incidence in clinical studies.

Although the current in vitro model demonstrates overall antimicrobial efficacy, there are other dressing features that are important in surgical wound management, including duration of wear, exudate management, pain on removal, wear comfort, and flexibility. Such features contribute to the overall performance of the dressing and can only be fully assessed in the clinical setting.

## Conclusions

In conclusion, the findings from the current study indicate that the ISEB SCD has improved antimicrobial performance in an in vitro direct inoculation simulated wound fluid model against planktonic phenotype SSI-causing bacteria compared with other silver-containing SCDs.
